# Autologous Collagen-Induced Chondrogenesis (ACIC) for Osteochondral Lesions of the Talus: A Systematic Review and Meta-Analysis

**DOI:** 10.3390/medicina62101826

**Published:** 2026-09-22

**Authors:** Elena Artioli, Antonio Mazzotti, Cesare Faldini

**Affiliations:** 1 Department of Biomedical and Neuromotor Sciences (DIBINEM), Alma Mater Studiorum University of Bologna, 40123 Bologna, Italy; antonio.mazzotti@ior.it (A.M.); cesare.faldini@ior.it (C.F.); 2 1st Orthopaedics and Traumatologic Clinic, IRCCS Istituto Ortopedico Rizzoli, 40136 Bologna, Italy

**Keywords:** osteochondral lesions of the talus, cartilage, ankle, ACIC

## Abstract

*Background and Objectives*: Autologous Collagen-Induced Chondrogenesis (ACIC) is a regenerative technique for treating osteochondral lesions that combines microfracture with the application of an injectable atelocollagen scaffold over the defect. This scaffold stabilizes the marrow clot and promotes the retention of mesenchymal stem cells at the repair site. Its use for Osteochondral Lesions of the Talus (OLTs) has increased in recent years, with several studies reporting favorable clinical outcomes. However, the available evidence has not yet been comprehensively synthesized. The aim of this systematic review and meta-analysis was to evaluate the clinical outcomes of ACIC for the treatment of OLTs. *Materials and Methods*: This study was conducted according to the Preferred Reporting Items for Systematic Reviews and Meta-Analyses (PRISMA) guidelines. Studies evaluating ACIC for the treatment of OLTs were systematically identified and selected. Data regarding patient characteristics, surgical technique, and clinical outcomes were extracted and analyzed. *Results*: Five studies were included in the systematic review, comprising 97 patients (50 males and 47 females) with a mean age of 40.9 years (range, 25.6–48.6 years). Meta-analysis demonstrated a significant improvement in functional outcomes, with an estimated Mean Difference (MD) in American Orthopaedic Foot & Ankle Society (AOFAS) scores of 26.54 points (95% CI, 17.34–35.74; *p* < 0.001). Pain outcomes also significantly improved, with a pooled MD in Visual Analogue Scale (VAS) scores of −3.72 points (95% CI, −4.44 to −3.01; *p* < 0.001). *Conclusions*: ACIC for OLTs provides consistent improvements in pain and functional outcomes across the available studies. The finding supported by the available data is that postoperative scores improved in small, predominantly uncontrolled cohorts, but the incremental benefit attributable to ACIC over conventional microfracture alone remains uncertain. Therefore, further adequately powered prospective studies directly comparing ACIC with microfracture alone are needed to better establish its clinical efficacy.

## 1. Introduction

Osteochondral Lesions of the Talus (OLTs) are a common cause of persistent ankle pain and functional limitation, particularly in young and active individuals [1,2]. Among the available surgical options [3], microfracture remains one of the most widely adopted first-line treatments for small- to medium-sized lesions because of its technical simplicity and satisfactory short-term outcomes [4,5]. By penetrating the subchondral bone, microfracture stimulates the release of bone marrow-derived Mesenchymal Stem Cells (MSCs), which possess chondrogenic potential and promote fibrocartilaginous repair through the secretion of trophic and anti-inflammatory factors [6].

Despite its widespread use, microfracture has intrinsic limitations, as the repair tissue consists predominantly of fibrocartilage, which has inferior biomechanical properties and durability compared with native hyaline cartilage [7]. To improve the biological environment for cartilage repair, several scaffold-based strategies have been developed to stabilize the marrow clot, retain MSCs within the defect, and support chondrogenesis [8,9]. Although encouraging clinical results have been reported, the implantation of solid scaffolds may be technically demanding in the ankle because of the limited surgical access and, in some cases, requires malleolar osteotomy to achieve adequate exposure of the lesion [8,10].

Autologous Collagen-Induced Chondrogenesis (ACIC) combines microfracture with the application of an injectable atelocollagen scaffold over the microfractured defect to stabilize the marrow clot and enhance MSC retention at the repair site [11]. Atelocollagen is a highly purified, cell-free type I collagen in which the antigenic telopeptides have been removed from both ends of the collagen triple helix, markedly reducing its immunogenicity [12]. Mixed with fibrin glue, it can be delivered arthroscopically as an injectable scaffold that polymerizes in situ, conforming to the defect geometry while providing mechanical stability to the blood clot [13,14]. Compared with solid scaffolds, this injectable approach simplifies implantation and facilitates the treatment of lesions in surgically challenging locations.

The use of atelocollagen augmentation for OLTs has increased in recent years, with several studies reporting favorable outcomes [13,14,15,16,17]. However, the available evidence has not been comprehensively synthesized. Therefore, the purpose of this systematic review and meta-analysis was to evaluate the clinical outcomes of ACIC for the treatment of OLTs.

## 2. Materials and Methods

This study was conducted as a systematic review and meta-analysis in accordance with the Preferred Reporting Items for Systematic Reviews and Meta-Analyses (PRISMA) guidelines. The review aimed to evaluate the outcomes of ACIC for the treatment of OLTs. The review was not prospectively registered and no protocol was prepared. The PRISMA checklist is provided in Supplementary Table S1.

### 2.1. Search Strategy

A comprehensive literature search was performed in PubMed/MEDLINE, Scopus, and the Cochrane Library from database inception to 25 May 2026. The search strategy was developed to identify studies evaluating ACIC for the treatment of OLTs. The following search string was used in PubMed/MEDLINE: *(“Atelocollagen-Induced Chondrogenesis”[Title/Abstract] OR “Autologous Collagen-Induced Chondrogenesis”[Title/Abstract] OR ACIC[Title/Abstract] OR atelocollagen[Title/Abstract]) AND (ankle[Title/Abstract] OR talus[Title/Abstract] OR “ankle joint”[Title/Abstract] OR “osteochondral lesion of the talus”[Title/Abstract] OR OLT[Title/Abstract]) AND (cartilage[Title/Abstract] OR chondrogenesis[Title/Abstract] OR “cartilage repair”[Title/Abstract] OR microfracture[Title/Abstract]).*

The following search string was used in Cochrane Library: ((“Atelocollagen-Induced Chondrogenesis” OR “Autologous Collagen-Induced Chondrogenesis” OR ACIC OR atelocollagen):ti,ab,kw) AND (ankle OR talus OR “ankle joint” OR “osteochondral lesion of the talus” OR OLT):ti,ab,kw AND (cartilage OR chondrogenesis OR “cartilage repair” OR microfracture):ti,ab,kw.

The following search strategy was used in Scopus: *TITLE-ABS-KEY((“Atelocollagen-Induced Chondrogenesis” OR “Autologous Collagen-Induced Chondrogenesis” OR ACIC OR atelocollagen) AND (ankle OR “ankle joint” OR talus OR “osteochondral lesion of the talus” OR OLT) AND (cartilage OR chondrogenesis OR “cartilage repair” OR microfracture)).*

In addition, the reference lists of all included studies and relevant review articles were manually screened to identify any additional eligible publications.

### 2.2. Eligibility Criteria

Studies were considered eligible if they met the following criteria:

1. Included patients with OLTs treated surgically;
2. Investigated ACIC as the primary intervention, defined as bone marrow stimulation (microfracture or drilling) combined with an injectable atelocollagen scaffold;
3. Reported preoperative and postoperative clinical and/or radiological outcomes;
4. Included human subjects.

Studies were excluded if they:

1. Investigated lesions of anatomical sites other than the ankle;
2. Evaluated surgical techniques other than ACIC or presented mixed results not separable from ACIC;
3. Were animal, cadaveric, biomechanical, or in vitro studies;
4. Were review articles, editorials, technical notes, conference abstracts, or expert opinions without original clinical data.

No restrictions on publication date were applied. Only studies published in English were considered eligible for inclusion.

### 2.3. Study Selection and Data Extraction

After duplicate removal using Mendeley reference management software, two reviewers independently screened all retrieved titles and abstracts for eligibility. Studies considered potentially relevant were subsequently assessed through a full-text review. Any disagreements regarding study eligibility were resolved through discussion and consensus between the reviewers. The study selection process was documented using a PRISMA flow diagram.

The following variables were extracted from each included study:

- Authors and year of publication;
- Study design and level of evidence (LoE);
- Sample size and patient demographics (age and sex);
- Lesion characteristics (size and grading);
- Description of the surgical technique;
- Follow-up duration: the latest postoperative assessment available was selected for the analysis, without imposing a minimum follow-up duration;
- Clinical outcome measures;
- Complications and revision procedures.

### 2.4. Risk of Bias

Risk of bias was independently assessed by two reviewers. Randomized controlled trials were evaluated using the Cochrane Risk of Bias 2 (RoB 2) tool. Comparative non-randomized studies were assessed using the Risk Of Bias In Non-randomized Studies of Interventions (ROBINS-I) tool. Retrospective case series were assessed using the Joanna Briggs Institute (JBI) Critical Appraisal Checklist for Case Series. Each study was classified as low, moderate, serious, or critical risk of bias according to the corresponding methodological framework.

### 2.5. Statistical Analysis

Continuous variables were summarized as mean and standard deviation, while categorical variables were reported as counts and percentages. For studies reporting Visual Analogue Scale (VAS) on a 0–100 scale, values were linearly converted to a 0–10 scale by dividing scores by 10 to ensure consistency across studies.

Meta-analyses were performed when at least two studies reported sufficient preoperative and postoperative data. The analysis was carried out using the mean difference (MD) as the effect size measure. Given that the measurements were obtained from the same patients, the variance calculation should be based on the standard deviation of the paired change. However, as the included studies did not report change-score standard deviations, within-patient correlations, or alternative methods for accounting for repeated measurements, the meta-analysis was conducted assuming a within-patient correlation of r = 0. Sensitivity analyses were then performed using a range of plausible correlation coefficients to assess the robustness of the results. A random-effects model was fitted to the data. Between-study variance (tau^2^) was estimated using the restricted maximum likelihood (REML) method. Heterogeneity was assessed using Cochran’s Q test and quantified with the I^2^ statistic. In addition, 95% prediction intervals were calculated to estimate the expected range of true effects in future settings. Heterogeneity was interpreted according to conventional thresholds: low (I^2^ < 25%), moderate (25–50%), substantial (50–75%), and considerable (>75%). Statistical significance was set at *p* < 0.05 (two-sided). All analyses were performed using Jamovi with the MAJOR meta-analysis module (The Jamovi Project, version 2.3, 2022).

## 3. Results

### 3.1. Search Results

The literature search identified a total of 24 records. After the removal of duplicate articles, 14 studies remained for screening. Following title screening, 10 articles were considered potentially eligible and underwent abstract review. After assessment of abstracts, 7 studies were selected for a full-text evaluation. The full-text review resulted in the exclusion of 2 studies that did not meet the predefined eligibility criteria. Ultimately, 5 studies were included in the qualitative synthesis and quantitative meta-analysis. The study selection process is summarized in the PRISMA flow diagram (Figure 1).

### 3.2. Study Design and Risk of Bias

Among the included articles, 3 were retrospective case series (LoE 4): one was a retrospective comparative case series (LoE 3), and one was a randomized controlled trial (LoE 1). The quality of the included study is represented in Figure 2 and Table 1.

### 3.3. Population

A total of 97 patients were included in this systematic review, comprising 50 males and 47 females. Four articles reported the mean age at surgery, with a weighted mean age of 40.9 (range 25.6–48.6) years. Lesion size varied across studies, ranging from small to large defects. Mean lesion size ranged from 76.6 ± 33.6 mm^2^ to 310 ± 60 mm^2^. Three studies reported lesion grading and classification systems. Two authors used the ICRS classification and included lesions ranging from grade II to IV, whereas Gatti et al. [13] used the Hepple classification and included lesions graded II–IV (Table 2).

### 3.4. Surgical Technique

The surgical technique was largely standardized across the included studies and was performed as originally described by Volpi et al. [15]. A thigh tourniquet was applied and standard anteromedial and anterolateral arthroscopic portals were established. After routine debridement of the lesion, microfractures were performed. The tourniquet was then released, and fluid irrigation was discontinued to prevent dilution of the injectable scaffold. The atelocollagen matrix was mixed with synthetic fibrin glue at a 1:1 ratio immediately before application and delivered arthroscopically using a dedicated dual-syringe system. After injection, the material was evenly distributed over the defect and allowed to polymerize in situ for approximately 5–8 min before completion of the procedure [15].

Most authors used the injectable atelocollagen scaffold CartiFill^TM^ (Sewon Cellontech, Co., Ltd., Seoul, Republic of Korea), whereas only one study employed ChondroFiller^®^ liquid (Meidrix Biomedicals GmbH, Esslingen am Neckar, Germany) [13]. The procedure was generally performed as an isolated intervention, except in the case series by Gatti et al., in which concomitant lateral ligament repair was performed in 17 of 21 patients [13]. Postoperative rehabilitation protocols showed considerable heterogeneity among the included studies, as summarized in Table 3.

### 3.5. Outcomes

The clinical and radiological outcomes of the ACIC techique reported in the included studies are summarized in Table 4. No complications were reported in any of the included studies.

#### 3.5.1. AOFAS

Four studies comprising 76 patients reported preoperative and postoperative AOFAS scores. Effect sizes were calculated using the MD, as all studies used the same measurement scale (AOFAS). The observed mean differences ranged from 19 to 35.75, with all estimates indicating an increase in postoperative AOFAS scores compared to baseline. The estimated average MD was 26.54 (95% CI: 17.34 to 35.74), showing a significant improvement following treatment. Substantial heterogeneity was observed across studies (I^2^ = 70%), indicating considerable variability in treatment effects. Overall, the increase in AOFAS scores was statistically significant (*p* < 0.001) (Figure 3).

A leave-one-out sensitivity analysis was performed to evaluate the robustness of the pooled effect and to identify potentially influential studies (Figure 4). Sequential exclusion of each study resulted in pooled MDs ranging from 21.31 (95% CI: 14.72–27.90) to 31.17 (95% CI: 22.45–39.90), with all pooled estimates remaining statistically significant. The exclusion of Tan et al. [17] resulted in the largest change in the pooled estimate, which decreased to an MD of 21.31 (95% CI: 14.72–27.90), and substantially reduced heterogeneity from I^2^ = 75.5% to I^2^ = 21.1%. Tan et al. (2024) [17] also showed the highest studentized residual (rstudent = 2.293), DFFITS (1.865), and Cook’s distance (1.061), indicating a potentially influential effect on the pooled estimate and between-study heterogeneity. Exclusion of Lee et al. [14] also substantially reduced heterogeneity (I^2^ = 31.3%), although its studentized residual did not exceed 2 (rstudent = −1.786). In contrast, removal of Volpi et al. [15] or Usuelli et al. [16] produced smaller changes in the pooled effect and did not reduce heterogeneity. Overall, the leave-one-out analysis confirmed that the direction and statistical significance of the treatment effect were robust to the exclusion of individual studies, although Tan et al. [17] appeared to contribute substantially to the observed heterogeneity.

The sensitivity analyses showed that the pooled effect estimate remained virtually unchanged across the range of plausible correlation coefficients (Table 5).

#### 3.5.2. VAS

Five studies comprising 97 patients reported preoperative and postoperative VAS scores. Effect sizes were calculated using the MD, as all studies used the same measurement scale (VAS). The observed mean differences ranged from 2.90 to 5.00, with all estimates indicating a reduction in postoperative VAS scores compared to baseline. The estimated average MD was −3.72 (95% CI: −3.01 to −4.44), showing a significant improvement following treatment. Moderate heterogeneity was observed across studies (I^2^ = 46%), indicating some variability in treatment effects. Overall, the reduction in VAS scores was statistically significant (*p* < 0.001) (Figure 5).

A sensitivity analysis was performed to assess the robustness of the pooled effect estimate after exclusion of the study by Gatti et al., as this study included a concomitant procedure in 17 of 21 patients, representing a potential source of clinical heterogeneity [13]. After exclusion of this study, four studies comprising 76 patients remained in the analysis. The pooled mean difference was −4.07 (95% CI, −4.74 to −3.40), compared with −3.72 (95% CI, −4.44 to −3.01) in the primary meta-analysis. The direction and statistical significance of the pooled effect remained unchanged, with the pooled estimate indicating a significant reduction in postoperative VAS scores (*p* < 0.001). Heterogeneity decreased from moderate in the primary analysis (I^2^ = 46%) to none after exclusion of the study (I^2^ = 0%; Q-test *p* = 0.478). No evidence of outliers or undue influence was identified. Overall, these findings support the robustness of the primary meta-analytic results to the exclusion of the study by Gatti et al. [13] (Figure 6).

Sensitivity analyses using different assumptions for the within-patient correlation coefficient yielded pooled mean changes ranging from −3.78 to −3.84, with results remaining consistent with the primary analysis (Table 6).

## 4. Discussion

The principal finding of the present systematic review and meta-analysis is that atelocollagen augmentation combined with microfracture (ACIC technique) is associated with significant improvements in both functional outcomes and pain in patients with OLTs. Pooled analysis demonstrated a mean improvement of 26.5 points in the AOFAS score and a mean reduction of 3.7 points in the VAS score, both of which were statistically significant. These findings suggest that the ACIC technique provides favorable short- to mid-term clinical outcomes; however, as these results are based predominantly on retrospective and uncontrolled studies, the incremental benefit attributable to ACIC over conventional microfracture remains uncertain. Future studies should prioritize adequately powered prospective studies directly comparing ACIC with microfracture alone.

The only randomized controlled trial comparing microfracture alone with ACIC demonstrated superior cartilage repair in the ACIC group at two years [14]. Patients treated with atelocollagen achieved significantly higher MOCART scores, while second-look histological analysis revealed repair tissue rich in type II collagen with a hyaline-like appearance [14]. These findings are consistent with preclinical evidence showing that microfracture combined with atelocollagen results in superior histological cartilage repair compared with microfracture alone, with regenerated tissue more closely resembling native hyaline cartilage [12]. Nevertheless, both experimental and clinical evidence suggest that atelocollagen augmentation promotes more hyaline-like cartilage regeneration than microfracture alone [12,14]. Given that repair tissue quality is considered a major determinant of long-term durability, it is reasonable to hypothesize that these biological advantages may translate into improved long-term clinical outcomes. However, this hypothesis remains to be confirmed through studies with extended follow-up.

The observed improvement in AOFAS score exceeds the minimal clinically important difference (MCID) reported for foot and ankle procedures [18], and it is comparable to other surgical techniques [19,20,21]. Likewise, the pooled reduction in VAS score reflects substantial pain relief after surgery, supporting the clinical effectiveness of this biological augmentation strategy [22]. However, these comparisons should be considered indirect, as the AOFAS MCID was derived from patients with unstable ankle fractures, while the VAS threshold was derived from acute postoperative pain rather than chronic osteochondral talar lesions. Furthermore, the AOFAS is not a purely patient-reported outcome measure, and reliance on this score limits direct comparison with contemporary osteochondral talar lesions literature using validated patient-reported outcome measures.

Moderate-to-substantial heterogeneity was observed across the pooled analyses, particularly for AOFAS scores. This variability is likely related to differences in patient characteristics, lesion size, rehabilitation protocols, follow-up duration, and the inclusion of concomitant procedures in one study [13]. The follow-up duration varied across the included studies, and the latest postoperative assessment available from each study was used without imposing a minimum follow-up threshold. This approach maximized the available longitudinal information, although the variability in follow-up duration should be considered when interpreting the pooled results. However, the differences in follow-up duration may not have a substantial impact on the overall findings, as previous evidence from Tan et al. suggests that the greatest improvements in VAS and AOFAS scores occur within the first 3 months postoperatively, with only modest additional improvements reported up to 1 year postoperatively [17]. Moreover, all included studies consistently demonstrated improvements in both functional outcomes and pain, supporting the overall robustness of the findings.

No complications were reported across the included studies, suggesting a favorable safety profile. However, these findings should be interpreted with some caution, given the relatively small overall sample of 97 patients and the predominantly retrospective nature of the available evidence. Larger prospective studies with longer follow-up will be valuable to further confirm the safety profile of the procedure.

The rationale for atelocollagen augmentation lies in addressing one of the main limitations of microfracture alone, namely the instability of the marrow clot. Following subchondral bone perforation, successful cartilage repair seems to depend on maintaining the clot and the contained MSCs within the defect. Injectable atelocollagen provides a three-dimensional scaffold, easy for arthroscopic positioning, that stabilizes the clot and may enhance cell retention during the early phases of repair. An in vitro study by Volpi et al. demonstrated that the presolid state of atelocollagen allows homogeneous distribution of both bone marrow MSCs and chondrocytes throughout the scaffold, providing further biological support for its use [15]. The clot containment may be particularly relevant for larger (>1 cm^2^) or uncontained lesions, where it is more challenging, as suggested by the literature [13,23].

Unfortunately, some limitations must be acknowledged. First, the certainty of the available evidence is low, given that it is based on a limited number of studies with relatively small sample sizes, most of which were retrospective case series with meaningful risk of bias and without comparative control groups. Consequently, the extent to which the observed clinical improvements are attributable to atelocollagen augmentation rather than microfracture itself cannot be definitively established.

Furthermore, the included studies involved heterogeneous lesion characteristics, including differences in lesion size, containment, cystic involvement, and previous surgical treatment. This heterogeneity limits the clinical comparability of the included populations and may have contributed to the variability in reported outcomes. In addition, rehabilitation protocols were not consistently standardized across studies and may substantially influence functional outcomes. Therefore, the observed improvements should not be attributed solely to the surgical technique without considering the potential contribution of differences in postoperative rehabilitation.

Finally, no studies have yet reported long-term clinical outcomes.

## 5. Conclusions

ACIC provides consistent improvements in pain relief and functional outcomes in the treatment of OLTs across the available studies. However, the current evidence remains limited, with a small number of published studies, relatively small patient cohorts, short follow-up periods, and a lack of high-quality comparative trials. The finding supported by the available data is that postoperative scores improved in small, predominantly uncontrolled cohorts, but the incremental benefit attributable to ACIC over microfracture alone remains uncertain. Further prospective studies with larger populations and longer follow-up are required to confirm the long-term safety and durability of the results and to determine the true added clinical benefit of atelocollagen augmentation compared with microfracture alone.

## Figures and Tables

**Figure 1:**
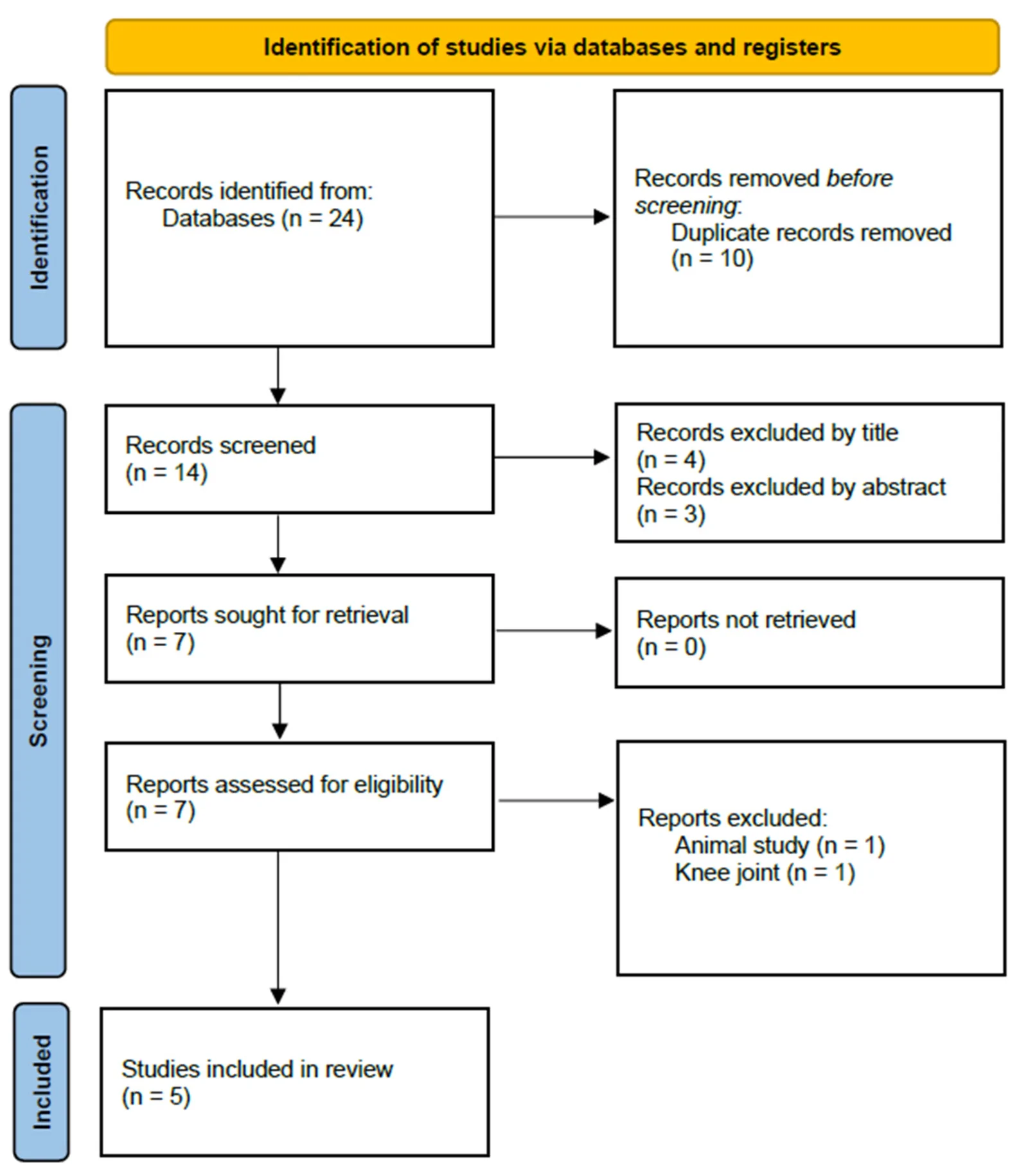
PRISMA flow diagram.

**Figure 2:**
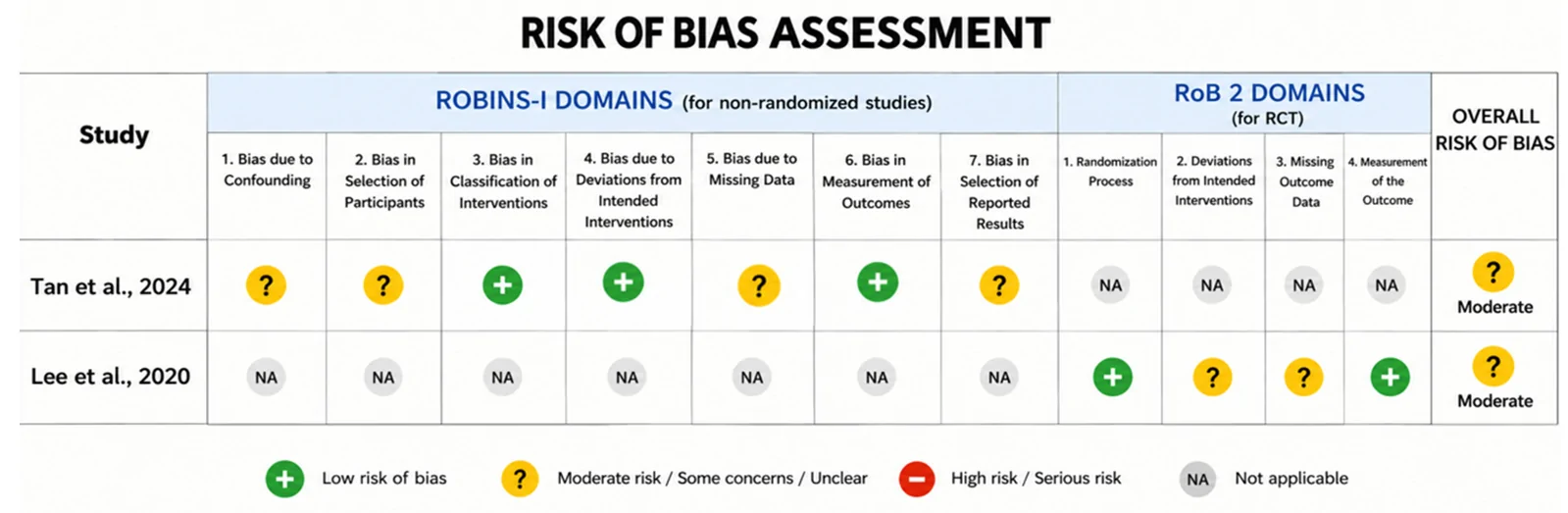
Risk-of-bias assessment through ROBINS-I for a comparative non-randomized study [17] and RoB 2 for RCT [14].

**Figure 3:**
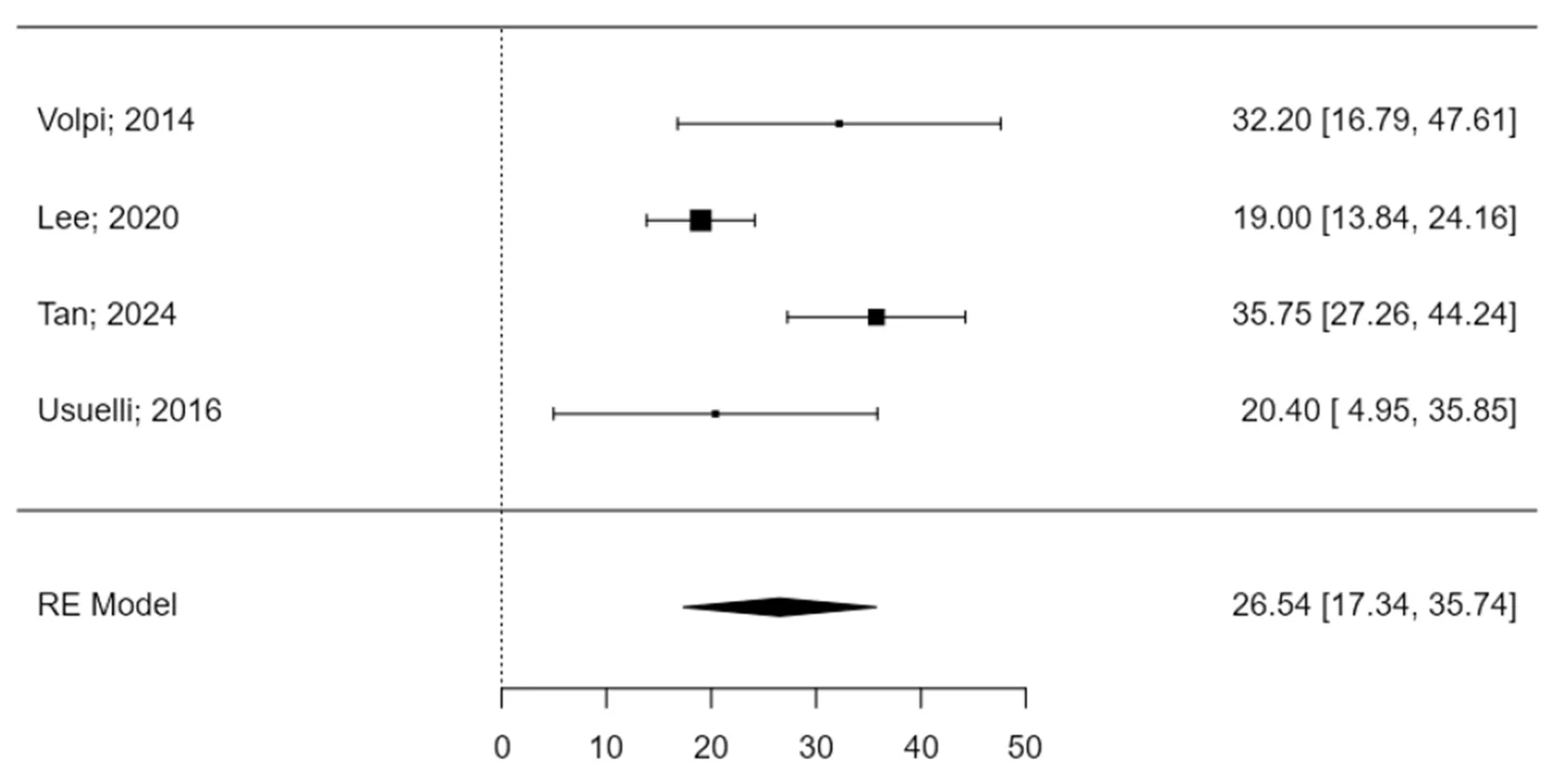
Forest plot of MD in AOFAS scores across the included studies [14,15,16,17].

**Figure 4:**
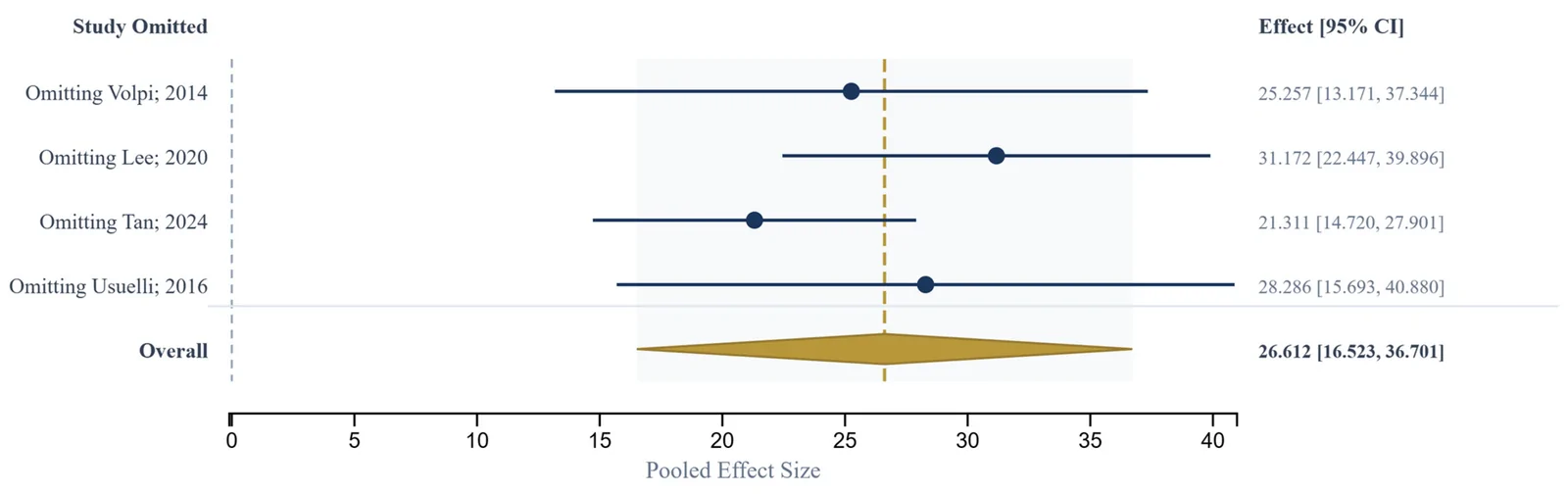
Forest plot of the leave-one-out sensitivity analysis [14,15,16,17].

**Figure 5:**
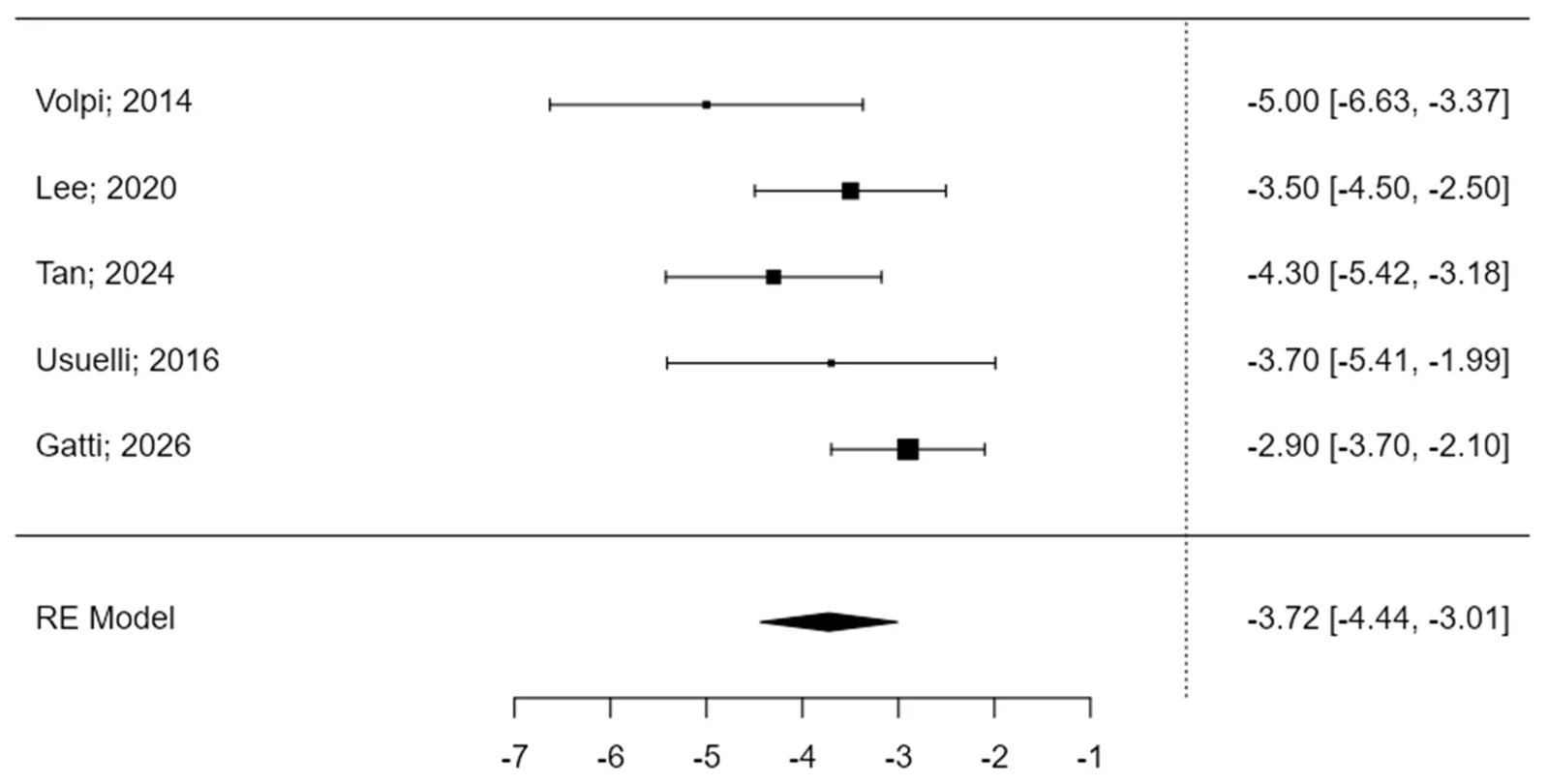
Forest plot of MD in VAS scores across the included studies [13,14,15,16,17].

**Figure 6:**
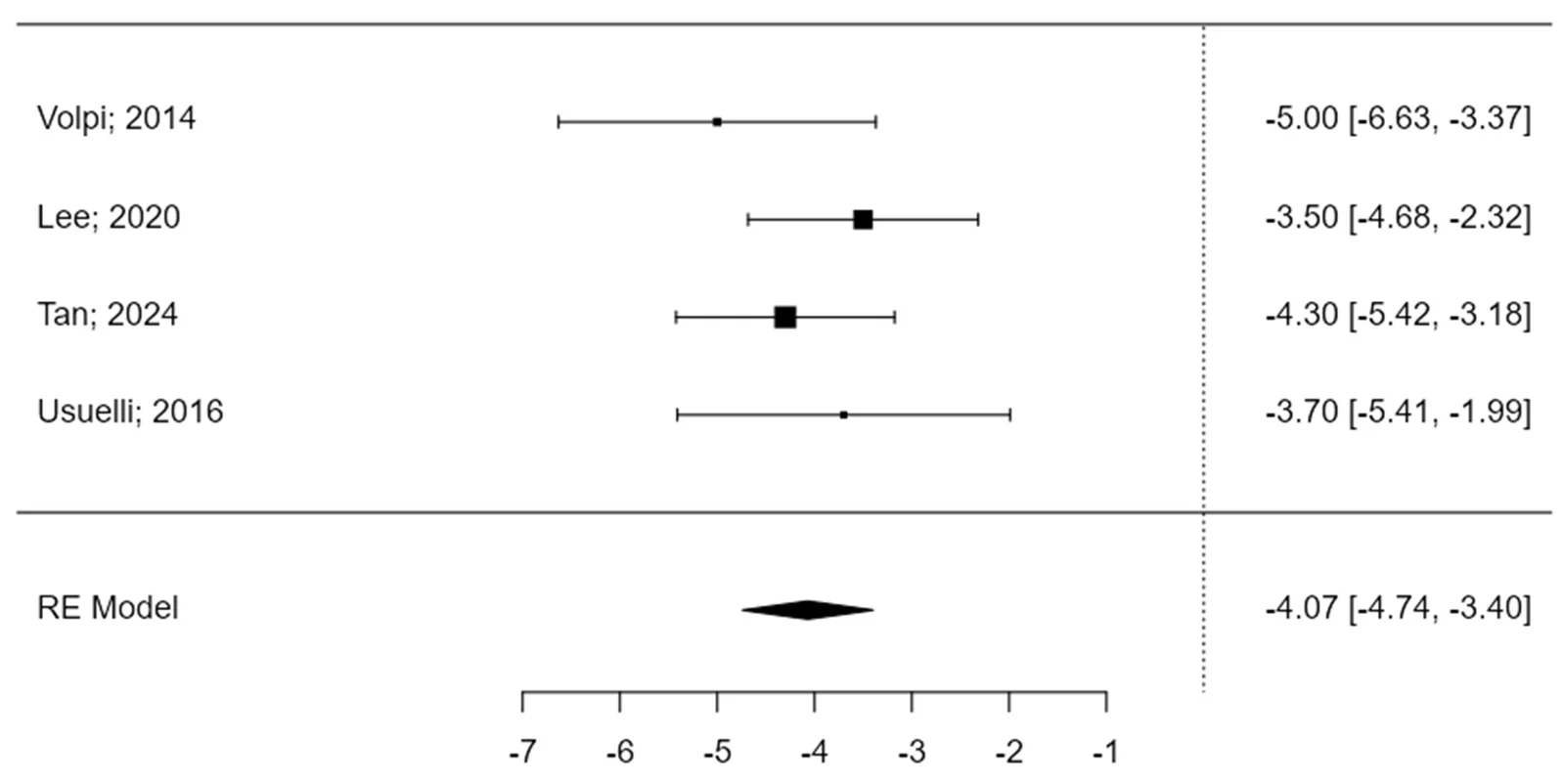
Forest plot of MD in VAS scores across the studies [14,15,16,17] after the exclusion of Gatti et al. [13].

**Table 1:** Risk of bias of retrospective case series assessed using the JBI Critical Appraisal Checklist for Case Series. Y: yes, N: no, U: unclear.

	Volpi et al., 2014 [15]	Usuelli et al., 2016 [16]	Gatti et al., 2026 [13]
Were there clear criteria for inclusion in the case series?	Y	Y	Y
Was the condition measured in a standard, reliable way for all participants included in the case series?	Y	Y	Y
Were valid methods used for identification of the condition for all participants included in the case series?	Y	Y	Y
Did the case series have consecutive inclusion of participants?	U	Y	U
Did the case series have complete inclusion of participants?	U	Y	U
Was there clear reporting of the demographics of the participants in the study?	Y	Y	Y
Was there clear reporting of clinical information of the participants?	Y	Y	Y
Were the outcomes or follow up results of cases clearly reported?	Y	Y	Y
Was there clear reporting of the presenting site(s)/clinic(s) demographic information?	U	U	U
Was statistical analysis appropriate?	Y	N	Y

**Table 2:** Population characteristics of the included study. NR: not reported.

Author and Year	Type of Study	LoE	N°	Sex M:F	Mean Age Years ± SD	Lesion Size mm^2^	Classification and Grade
Volpi; 2014 [15]	Retrospective case series	4	5	3:2	25.6 ± 6.6	310 ± 60	ICRS III–IV
Lee; 2020 [14]	Randomized controlled trial	1	31	15:16	35 ± 15.7	96.8 ± 71.9	ICRS II–IV
Tan; 2024 [17]	Retrospective comparative case series	3	31	12:19	48.6 ± 15.1	<150	NR
Usuelli; 2016 [16]	Retrospective case series	4	9	6:3	37.4 ± 10	210 ± 90	NR
Gatti; 2026 [13]	Retrospective case series	4	21	14:7	NR	76.6 ± 33.6	Hepple II–IV

**Table 3:** Surgical technique; w = weeks, mo = months, WB = weight bearing, ROM = range of motion.

Author and Year	Surgical Procedure	Additional Procedure	Scaffold	Postoperative Care	Complications
Volpi; 2014 [15]	ACIC		CartiFill^TM^	No boot or cast	0
Lee; 2020 [14]	ACIC		CartiFill^TM^	4 w posterior splint; 2 w partial WB; full WB after 4 w; jog after 3 mo	0
Tan; 2024 [17]	ACIC		CartiFill^TM^	2 w backslab then 4 w walker boot; immediate ROM; 6 w no-WB	0
Usuelli; 2016 [16]	ACIC		CartiFill^TM^	6 w no-WB; 2 w Limited ROM; After 6–8 w proprioceptive exercises and low-impact sports; Full activity after 3–4 mo	0
Gatti; 2026 [13]	ACIC	Ligament repair in 17/21 patients	ChondroFiller^®^	3 w non-WB then progressive WB in walker boot; immediate ROM if no ligament repair	0

**Table 4:** Clinical and radiological outcomes.

Author and Year	N°	AOFAS PRE Mean ± SD	AOFAS POST Mean ± SD	VAS PRE Mean ± SD	VAS POST Mean ± SD	Others	Follow Up Months
Volpi; 2014 [15]	5	53.8 ± 15.1	86 ± 9	6.6 ± 1.1	1.6 ± 1.5	Tegner: pre 6 ± 3.1; post 5.1 ± 1.3	6
Lee; 2020 [14]	31	72.23 ± 11.85	91.23 ± 8.62	5.2 ± 2	1.7 ± 2	MOCART 64.49 ± 18.27	24
Tan; 2024 [17]	31	46.8 ± 15.9	82.55 ± 18.12	6 ± 2.1	1.7 ± 2.4		12
Usuelli; 2016 [16]	9	51.4 ± 11.6	71.8 ± 20.6	6.9 ± 1.8	3.2 ± 1.9	MOCART 51.7 ± 16.6	12
Gatti; 2026 [13]	21			3.2 ± 1.8	0.3 ± 0.5		37.4 ± 20.7

**Table 5:** Sensitivity analysis according to different assumed within-patient correlation coefficients for the AOFAS score.

Assumption	Pooled Mean Change	95% CI	I^2^
r = 0.3	26.76	16.79–36.74	80.7%
r = 0.5	26.81	16.80–36.81	85.9%
r = 0.7	26.84	16.74–36.94	91.2%

**Table 6:** Sensitivity analysis according to different assumed within-patient correlation coefficient for VAS score.

Assumption	Pooled Mean Change	95% CI	I^2^
r = 0.3	−3.78	−4.51 to −3.06	58.4%
r = 0.5	−3.82	−4.53 to −3.10	67.3%
r = 0.7	−3.84	−4.53 to −3.16	76.7%

## Data Availability

The original contributions presented in this study are included in the article/Supplementary Material. Further inquiries can be directed to the corresponding author.

## Supplementary Materials

The following supporting information can be downloaded at: https://www.mdpi.com/article/10.3390/medicina62101826/s1. Table S1: PRISMA 2020 Checklist.

## Abbreviations

The following abbreviations are used in this manuscript:

ACIC	Autologous collagen-induced chondrogenesis
OLT	Osteochondral lesions of the talus
MSC	Mesenchymal stem cell
VAS	Visual Analogue Scale
LoE	Level of evidence
AOFAS	American Orthopaedic Foot & Ankle Society score
